# A Synthetic Small Molecule, LGM2605: A Promising Modulator of Increased Pro-Inflammatory Cytokine and Osteoclast Differentiation by *Aggregatibacter actinomycetemcomitans* Cytolethal Distending Toxin

**DOI:** 10.3390/dj12070195

**Published:** 2024-06-26

**Authors:** Taewan J. Kim, Andrew S. MacElroy, Aleena Defreitas, Bruce J. Shenker, Kathleen Boesze-Battaglia

**Affiliations:** 1 Department of Periodontics, School of Dental Medicine, University of Pennsylvania, Philadelphia, PA 19104, USA; taewank@upenn.edu; 2 Department of Basic and Translational Sciences, School of Dental Medicine, University of Pennsylvania, Philadelphia, PA 19104, USA; amacelro@upenn.edu (A.S.M.); aleenad@upenn.edu (A.D.); shenker@upenn.edu (B.J.S.)

**Keywords:** localized aggressive periodontitis, molar/incisor pattern, flaxseed, anti-inflammation, osteoclast, small molecule therapeutic

## Abstract

Our research explores the interplay between *Aggregatibacter actinomycetemcomitans* (*Aa*) cytolethal distending toxin (Cdt) and the host’s inflammatory response in molar/incisor pattern periodontitis (MIPP). Cdt disrupts phosphatidylinositol-3,4,5-triphosphate (PIP3) signaling, influencing cytokine expression through canonical and non-canonical inflammasome activation as well as nuclear factor-κB (NF-κB) activation, leading to inflammation in MIPP. THP-1 differentiated macrophages (TDMs) exposed to Cdt exhibited an upregulation of pro-inflammatory genes and subsequent cytokine release. We analyzed the ability of a small molecule therapeutic, LGM2605, known for its anti-inflammatory properties, to reduce pro-inflammatory gene expression and cytokine release in Cdt-exposed and *Aa*-inoculated TDMs. LGM2605’s mechanism of action involves inhibiting NF-κB while activating the Nrf2–transcription factor and antioxidants. Herein, we show that this small molecule therapeutic mitigates Cdt-induced pro-inflammatory cytokine expression and secretion. Our study also further defines Cdt’s impact on osteoclast differentiation and maturation in MIPP. Cdt promotes increased TRAP+ cells, indicating heightened osteoclast differentiation, specific to Cdt’s phosphatase activity. Cathepsin K levels rise during this process, reflecting changes in TRAP distribution between control and Cdt-treated cells. Exploring LGM2605’s effect on Cdt-induced osteoclast differentiation and maturation, we found TRAP+ cells significantly reduced with LGM2605 treatment compared to Cdt alone. Upon LGM2605 treatment, immunocytochemistry revealed a decreased TRAP intensity and number of multinucleated cells. Moreover, immunoblotting showed reduced TRAP and cathepsin K levels, suggesting LGM2605’s potential to curb osteoclast differentiation and maturation by modulating inflammatory cytokines, possibly involving Nrf2 activation. In summary, our research reveals the intricate connections between Cdt, pro-inflammatory cytokines, and osteoclast differentiation, offering novel therapeutic possibilities for managing these conditions.

## 1. Introduction

Periodontal diseases encompass a group of multifactorial inflammatory conditions that affect the supporting structures of the teeth, including the gingiva, periodontal ligament, and alveolar bone [1,2]. Among these, molar/incisor pattern periodontitis (MIPP), formerly known as localized aggressive periodontitis (LAP), stands out as a distinct and severe form of periodontal disease characterized by rapid tissue destruction without apparent poor oral hygiene and visible clinical inflammation [1]. The etiology of MIPP has long intrigued researchers, as it is localized, and its aggressive nature suggests the involvement of microbial dysbiosis and uncontrolled host response [1,2,3].

One microbial factor that has garnered significant attention in the context of MIPP is *Aggregatibacter actinomycetemcomitans* (*Aa*). Among toxins that are produced by *Aa*, cytolethal distending toxin (Cdt) plays a significant role in distorting the host response [4]. It acts as a phosphatidylinositol-3,4,5-triphosphate (PIP3) phosphatase and causes a PI-3K signaling blockade which leads to a decrease in phagocytic response, exacerbates inflammation, and neutralizes the immune response [5,6,7]. Also, there is a higher occurrence of Cdt-expressing *Aa* in MIPP patients [8]. This suggests a potential role for Cdt in both the pathogenesis and clinical manifestation of MIPP. However, the precise mechanisms through which Cdt contributes to MIPP remain incompletely understood and its role in alveolar bone loss underexplored.

Osteoclasts (OC), specialized cells responsible for bone resorption, play a crucial role in maintaining the delicate equilibrium between bone formation and breakdown [9,10]. The elevated differentiation and activity of osteoclasts can result in unchecked bone loss, which is a characteristic feature of periodontitis [9,10]. While nuclear factor κB ligand (RANKL) and macrophage colony-stimulating factor (M-CSF) are well-established regulators of osteoclast differentiation, various inflammatory cytokines can also foster this process. For example, the pro-inflammatory cytokine interleukin-1β (IL-1β) prominently promotes osteoclast differentiation and bone resorption by enhancing RANKL expression [10,11]. Similarly, IL-1β indirectly facilitates tumor necrosis factor alpha (TNF-α)-induced osteoclastogenesis by upregulating RANKL expression in stromal cells, and directly promotes the differentiation of OC precursors through p38 mitogen-activated protein kinase (MAPK) signaling in the presence of adequate RANKL [9,10]. Optimal levels of RANKL are also necessary for IL-1α to induce the expression of osteoclast markers such as tartrate-resistant acid phosphatase (TRAP), cathepsin K, matrix metallopeptidase 9 (MMP9), and nuclear factor of activated T-cells, cytoplasmic 1 (NFATc1) [9,10,11]. Additionally, interleukin-1α can independently trigger osteoclast differentiation by inducing microphthalmia transcription factor (MITF) in bone marrow macrophages (BMMs) [9,11].

Moreover, interleukin-6 (IL-6) positively influences osteoclastogenesis by stimulating RANKL expression in osteoblasts (OB) and stromal cells [9]. In the IL-6 signaling cascade, signal transducer and activator of transcription 3 (STAT3) is activated by Janus kinases (JAKs), resulting in the expression of osteoclast markers [9,12]. On the contrary, TNF-α serves as a potent stimulator of bone resorption and plays a pivotal role in bone metabolism and inflammatory bone disorders [9,13,14]. It directly triggers the formation of TRAP+ multinucleated osteoclasts from osteoclast precursors in the presence of M-CSF and in the absence of RANKL by activating nuclear factor-κB (NF-κB) signaling [9,13,14]. TNF-α also induces RANK expression in osteoclast precursors [9,13,14] and may expedite RANKL-induced osteoclastogenesis through tumor necrosis factor receptor–associated factor (TRAF) 2/5 and MAPK activation in tumor necrosis factor receptor (TNFR) 1-mediated signaling, leading to NF-κB and activator protein 1 (AP-1) activation [9,14]. Furthermore, TNF-α may indirectly impact osteoclastogenesis by inducing the expression of M-CSF and RANKL in stromal cells, OB, and activated T cells [9,13].

The inflammatory markers previously mentioned, capable of impacting osteoclast formation, are elevated not only through Cdt intoxication but also due to *Aa* infection, potentially contributing to the generation of osteoclasts and subsequent bone resorption [9]. Animal studies have confirmed that *Aa* triggers osteoclast formation, leading to subsequent bone loss [11]. In humans, individuals testing positive for *Aa* exhibit notably greater periodontal bone loss compared to *Aa*-negative individuals, highlighting *Aa*’s significant influence on alveolar bone loss linked to periodontal disease [12]. Consequently, Cdt and/or *Aa* may have a more intricate role in establishing an environment conducive to bone loss that contributes to MIPP pathogenesis.

Therapeutic strategies for addressing the host hyper-response observed in MIPP patients remain limited. Sub-antimicrobial doses of doxycycline, extensively studied for chronic periodontitis, act as a host modulatory agent [2,13]. Steroidal and non-steroidal anti-inflammatory drugs, commonly used as host modulatory pharmacological agents, have limitations due to their side effects, restricting their prolonged usage [3]. Consequently, alternative agents for treating inflammatory diseases are under development, with small molecules emerging as a promising avenue [3]. One such example is secoisolariciresinol diglucoside (SDG), a natural lignan recognized for its potent antioxidant and anti-inflammatory properties. Its synthetic equivalent, LGM2605, mimics these effects and has shown promise in various inflammatory-related diseases [14,15,16,17,18,19]. In conditions like asbestos-induced lung disease, excessive phagocytosis-related inflammation in retinal epithelium, radiation-induced lung vascular damage, and chronic neuroinflammation, LGM2605 resulted in a reduction in cellular toxicity, inflammation, and oxidative stress [15,16,17,18,20,21,22,23]. Considering these encouraging outcomes in addressing inflammatory conditions, the application of LGM2605 in MIPP holds potential for novel avenues in prevention and treatment. Our study elucidates the role of Cdt in macrophage and osteoclast differentiation, thereby advancing our understanding of MIPP pathogenesis. Additionally, we evaluated LGM2605 as a small molecule therapeutic candidate to alleviate Cdt-induced increased pro-inflammatory cytokine and osteoclast differentiation and maturation.

## 2. Materials and Methods

### 2.1. Materials and Reagents

Synthetic secoisolariciresinol diglucoside (SDG) (referred to as LGM2605 in the literature) was supplied by LignaMed LLC and met all batch analytical criteria including 1H NMR and HPLC/MS analysis. The compound was a white solid with >97% purity and a 51/49 diastereomeric ratio. Antibodies used for the experiments are listed in Table 1 with concentrations used for Western blot and immunocytochemistry.

### 2.2. Cellular Models

THP-1 cell-derived macrophages (TDM): The THP-1 cell line, derived from human acute monocytic leukemia and acquired from ATCC (Manassas, VA, USA), was cultured in RPMI1640 supplemented with 10% FBS, 1 mM sodium pyruvate, 20 μM 2-mercaptoethanol, and 2% penicillin–streptomycin at 37 °C with 5% CO_2_ in a humidified environment. To induce macrophage differentiation, cells were seeded on 35 mm glass bottom dishes (MatTek; Ashland, MA, USA), 12-well cell culture plates (Thermo Scientific; Waltham, MA, USA), or 96-well cell culture plates (Thermo Scientific; Waltham, MA, USA), and treated with 50 ng/mL phorbol 12-myristate 13-acetate (PMA) for 48 h. Subsequently, the cells were washed and further incubated in a fresh medium for an additional 24 h prior to experimentation [7].

THP-1 cell-derived osteoclast (TDO): THP-1 cells were differentiated into macrophages on either 6-, 12-, or 24-well cell culture plates (Thermo Scientific; Waltham, MA, USA) by incubating cells in the presence of 100 ng/mL PMA for 2 days. Cells were then incubated with 50 ng/mL of RANKL and M-CSF for 6 days for osteoclast differentiation. Cells were replenished with fresh media + RANKL and M-CSF (PeperoTech, Cranbury, NJ, USA) with and without other treatments (Cdt and LGM2605) every 48 h [24].

### 2.3. Expression and Purification of Cdt, CdtB Mutants, and Cdt Holotoxin

The assembly and expression of the plasmid containing the cdt genes encoding the holotoxin (pUCAacdtABChis) is described in prior studies [25]. The plasmid was engineered to incorporate the cdt genes under the regulation of the lac promoter and subsequently introduced into *E. coli* DH5α. The transformed *E. coli* cultures were grown in 1 L of LB broth and induced with 0.1 mM of isopropyl β-D-1-thiogalactopyranoside for 2 h. Following induction, the bacterial cells were harvested, washed, and reconstituted in 50 mM Tris buffer (pH 8.0). After an overnight freezing step followed by thawing, the cells were subjected to sonication. The purification of the histidine-tagged peptide holotoxin was accomplished using nickel affinity chromatography, as described previously [25].

### 2.4. Treatment with LGM2605

For macrophage studies: TDM were pretreated with different concentrations of LGM2605 (50–200 μM) in media for 30 min prior to Cdt (50–500 ng/mL) stress induction or *Aa* (MOI of 1:10 or 1:100). 

For osteoclast studies: After 48 h of PMA treatment (high concentrations: 100 ng/mL) to induce differentiation, THP-1 was pretreated with different concentrations of LGM2605 (50–200 μM) in media before RANKL and M-CSF (50 ng/mL each) induced osteoclast differentiation with or without Cdt treatment in different concentrations [24]. Media and LGM2605 replenishment were conducted with and without Cdt treatment every 48 h. 

### 2.5. RNA Isolation and Gene Expression Analysis

Total RNA extraction was carried out utilizing the RNeasy Plus Mini Kit, followed by quantitative polymerase chain reaction (qPCR) analysis, as outlined in previous literature [16]. The concentration of total RNA was determined using a NanoDrop 2000 spectrophotometer (Thermo Fisher Scientific, Waltham, MA, USA). Subsequently, the reverse transcription of RNA into cDNA was performed utilizing the High Capacity RNA-to-cDNA Kit on a Veriti^®^ Thermal Cycler (Applied Biosystems, Thermo Fisher Scientific, Waltham, MA, USA). For qPCR, individual TaqMan^®^ Probe-Based Gene Expression Assays (Applied Biosystems, Thermo Fisher Scientific, Waltham, MA, USA) were employed, with specific assays selected (see Table 2). Each reaction well contained 50 ng of cDNA and was run on an Applied Biosystems QuantStudio 6 Flex Real-Time PCR System (Applied Biosystems, Thermo Fisher Scientific, Waltham, MA, USA). Gene expression levels were normalized to the housekeeping gene glyceraldehyde 3-phosphate dehydrogenase (GAPDH), and all data were normalized to control samples (untreated THP-1 macrophages) utilizing the ΔΔCT method, as previously outlined [16].

### 2.6. Bacteria and Growth Curve

*A. actinomycetemcomitans* strains, D7S-SA (wild-type Aa) and D7S-SA CHE001 (Cdt-deficient Aa mutant), were procured following previously established procedures [25]. Each strain was cultured on AAGM agar medium comprising 20 g of BBL trypticase soy agar (BD; Sparks, MD, USA) and 3 g of yeast extract (ThermoFisher; Waltham, MA, USA), and supplemented with 0.4% sodium bicarbonate and 0.8% dextrose [25]. Subsequent to incubating the bacteria on plates for either 24 or 48 h in a CO_2_ incubator at 37 °C, they were transferred into 10 mL of AAGM broth until reaching an OD600 close to 0.2. Bacterial cultures from various dilutions were plated at different time intervals on AAGM agar plates and further incubated for 24 h in a CO_2_ incubator at 37 °C [25]. OD600 measurements were conducted at each time point using a DU 650 Spectrophotometer (Beckman Coulter, Indianapolis, IN, USA).

### 2.7. Tartrate-Resistant Acid Phosphatase (TRAP) Staining

On the 6th day of osteoclast differentiation, the supernatants were aspirated and discarded. Cells underwent three washes with PBS, followed by treatment with a fixation solution (composed of citrate buffer with 60% acetone and 10% methanol) for 5 min at room temperature. Subsequent to fixation, the staining of TRAP was conducted using the TRACP & ALP Double-stain Kit (MK300; Takara Bio Inc., Kusatsu, Shiga, Japan), adhering to the manufacturer’s instructions. TRAP-positive cells were enumerated utilizing a Nikon TMS-F inverted microscope.

### 2.8. Western Blot Analysis

Cells were lysed in buffer; 20 mM Tris-HCl (pH 7.5), 150 mM NaCl, 1 mM EDTA, 1% NP-40, 1% sodium deoxycholate, and a protease inhibitor cocktail (ThermoFisher Scientific; Waltham, MA, USA). Subsequently, samples (15 μg) were subjected to separation on 4–10% SDS-PAGE gels and subsequently transferred onto PVDF membranes. The membranes were blocked using BLOTTO and then incubated with primary antibodies overnight (18 h) at 4 °C [25]. Following washing steps, the membranes were exposed to secondary antibodies conjugated with horseradish peroxidase [25]. Western blot signals were developed using chemiluminescence and quantified via digital densitometry (LiCor Biosciences; Lincoln, NE, USA) [25]. The normalization of each protein signal was performed relative to either actin or GAPDH.

### 2.9. Cytokine Analysis

Cytokines were quantified in the culture supernatants obtained from TDM challenges in the presence or absence of LGM2605 at concentrations specified in the figure legends. The collected culture supernatants, at specified time points indicated in the figure legends, underwent analysis via ELISA for IL-1β, IL-6, and TNF-α, utilizing commercially available DuoSet ELISA Kits (R&D Systems; Minneapolis, MN, USA), following the manufacturer’s protocols [7]. For each cytokine, the concentration in the supernatant was determined by reference to a standard curve.

### 2.10. Confocal Microscopy

The cells were fixed in 4% PFA for 15 mins, washed three times, and permeabilized in blocking solution containing 1% BSA, 1% normal donkey serum, and 0.1% saponin in PBS at room temperature for 1 h. Next, they were incubated with the primary antibody listed in the antibody table with concentration o/n at 4 °C in antibody solution (PBS with 1% BSA), washed in PBS three times, and the secondary antibody (1:1000) was added with nuclear staining (Hoechst 33298, 1:10,000). Samples were imaged using a Nikon A1R laser scanning confocal microscope with a PLAN APO VC 60× water (NA 1.2) objective. The intensity of 488 nm was measured and averaged with the number of cells in field. The number of nuclei per cell was also counted blindly in random field for each condition manually to determine the maturation of osteoclasts.

### 2.11. Cell Viability

TDM were treated for 24 h with LGM2605 in increasing concentrations (25–400 μM). After the treatment, cells were lifted with 0.05% trypsin–EDTA and treated with Vi-CELL XR reagents (Beckman Coulter; Sharon Hill, PA, USA) according to the manufacturer’s protocol. Cell viability was processed with the Vi-CELL XR Cell Viability Analyzer (Beckman Coulter) in experimental triplicate with technical triplicate. 

### 2.12. Statistical Analysis

Mean ± standard deviation was calculated within each experiment, using technical replicates as indicated in the figure legends. For direct comparison, student’s *t*-test was used with statistical significance at *p*-value < 0.05. In comparisons of the control and group of dosages, one-way ANOVA was used with statistical significance at *p*-value < 0.05. For comparison between groups of different dosages, two-way ANOVA was used with statistical significance at *p*-value < 0.05. For the convenience of the readers, the list of abbreviations is provided in Table 3.

## 3. Results

### 3.1. LGM2605 Treatment Downregulates Expression of Inflammatory Cytokines

**Figure 1:**
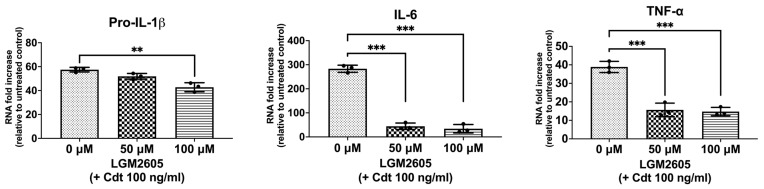
LGM2605 decreases Cdt-mediated upregulation of cytokine gene expression. TDM were pre-treated with increasing doses of LGM2605 for 30 min and 100 ng/mL of Cdt was added for 2 h. RNA was extracted and quantitative real-time PCR performed for pro-IL-1β, IL-6, and TNF-α and fold increase was calculated by normalization with a housekeeping gene (GAPDH) and compared to an untreated control (no Cdt and LGM2605 treatments). Data represent three independent experiments with technical triplicate. Statistical significance was determined using one-way ANOVA. ** denotes *p*-value < 0.01 and *** denotes *p*-value < 0.005 for Cdt only (0 uM LGM2605) vs. different doses of LGM2605.

Macrophage exposure to Cdt results in decreased PIP3 and the reduced phosphorylation of both glycogen synthase kinase 3 beta (GSK3β) and protein kinase B (Akt), leading to an increase in GSK3β kinase activity [5,7,26]. GSK3β, in turn, influences the association of the p65 subunit with the common co-activator (CBP), thereby influencing the expression of pro-inflammatory cytokines by modifying NF-κB activation [7]. To determine if Cdt treatment modulates cytokine expression, we treated TDM with the toxin (100 ng/mL) for 2 h and assessed the expression of pro-inflammatory genes (proIL-1β, IL-6, and TNF-α) using quantitative real-time PCR (Figure 1). There was a 57-fold increase in the expression of pro-IL-1β, a 283-fold increase in IL-6, and a 39-fold increase in TNF-α mRNA expression when compared to untreated TDM (control). These findings align with previous studies examining Cdt-mediated inflammatory mechanisms [7,27].

We then assessed LGM2605 for its ability to mitigate the Cdt-dependent enhancement of cytokine gene expression. Initially, TDM were pre-treated with LGM2605 (50 μM and 100 μM) for 30 min before exposure to Cdt (100 ng/mL for 2 h). Pro-IL-1β expression exhibited a small, but significant, reduction in the presence of LGM2605 to 52-fold (50 μM) and 43-fold (100 μM) compared to macrophages treated solely with Cdt (57-fold). In comparison, IL-6 exhibited a greater, and significant, reduction to 44-fold (at 50 μM LGM2605) and 33.7-fold (at 100 μM LGM2605) (*p* < 0.05) compared to Cdt-treated macrophages without LGM2605 (283-fold). Finally, TNF-α levels decreased significantly to 15-fold (at 50 μM LGM2605) and 16-fold (at 100 μM LGM2605) (*p* < 0.05) when compared to the macrophages treated only with Cdt (39-fold).

We also determined if LGM2605 was cytotoxic by treating TDM with increasing doses of LGM2605 (25, 50, 100, 200, and 400 μM) for 24 h. Adherent cells were lifted by trypsinization, and cell viability was assessed by using the Vi-CELL XR Cell Viability Analyzer. The Cdt-treated cells remained viable at all doses of LGM2605 (Figure S1).

### 3.2. LGM2605 Diminishes Cdt-Induced Pro-Inflammatory Cytokine Release

**Figure 2:**
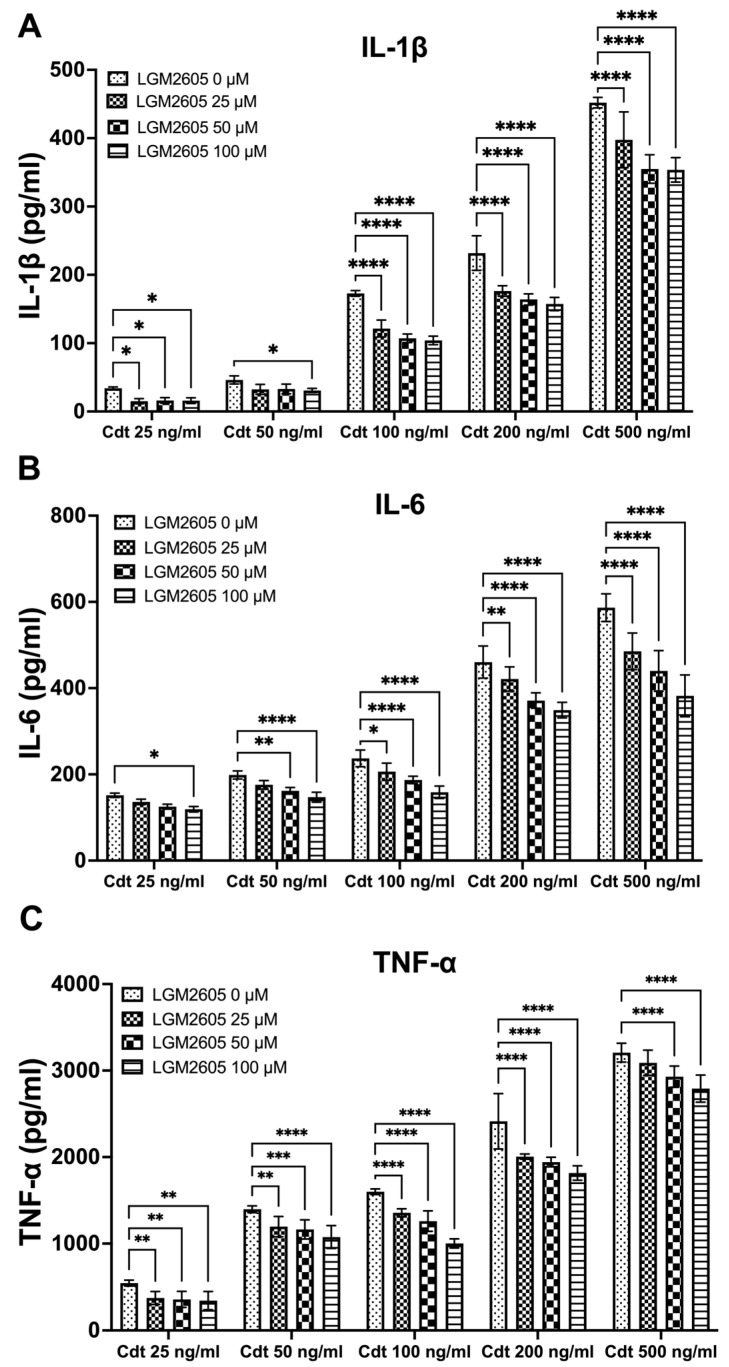
LGM2605 decreases Cdt-induced pro-inflammatory cytokines. TDM were pre-treated with increasing doses of LGM2605 for 30 min and Cdt (increasing concentrations) was added for 5 h. Supernatants were collected and analyzed by ELISA specific for IL-1β (Panel (**A**)), IL-6 (Panel (**B**)), and TNF-α (Panel (**C**)). Cytokines were undetectable in the absence of Cdt. Data represent three independent experiments with technical triplicate for ELISA. Statistical significance was determined using two-way ANOVA. * denotes *p*-value < 0.05, ** denotes *p*-value < 0.01, *** denotes *p*-value < 0.005, and **** denotes *p*-value < 0.0001 for 0 uM LGM2605 vs. a different dose of LGM2605 within same concentration Cdt-treated group.

We further investigated the impact of LGM2605 treatment on cytokine release. TMD were pretreated with varying doses (0, 25, 50, and 100 μM) of LGM2605 for 30 min, followed by the addition of increasing concentrations of Cdt (25, 50, 100, 200, and 500 ng/mL) for a 5 h period to promote cytokine release. The supernatants were collected, and levels of IL-1β (Figure 2A), IL-6 (Figure 2B) and TNF-α (Figure 2C) were determined by ELISA. Cdt treatment led to an increase in the release of pro-inflammatory cytokines at all concentrations studied (Figure 2, LGM2605 0 μM). Conversely, LGM2605 treatment reduced cytokine release when compared to Cdt exposure alone. Notably, for IL-1β, the most significant reduction in IL-1β release (40%) was observed in TDM treated with 100 ng/mL Cdt in combination with 100 μM LGM2605 (172 ± 6 pg/mL compared to 107 ± 6 pg/mL, Figure 2A). In the case of IL-6, the most substantial reduction in IL-6 released (40%) was observed in TDM treated with 500 ng/mL Cdt in combination with 100 μM LGM2605 (577 ± 45 pg/mL compared to 352 ± 22 pg/mL, Figure 2B). For TNF-α, the most pronounced reduction (40%) was noted in TDM treated with 100 ng/mL Cdt in combination with 100 μM LGM2605 (1593 ± 36 pg/mL compared to 985 ± 38 pg/mL, Figure 2C). Collectively, the 40% decrease in Cdt-mediated pro-inflammatory cytokines release is of particular significance in the context of a periodontium niche. In this microenvironment, structural components of the periodontium can be easily manipulated by inflammation.

### 3.3. LGM2605 Diminishes Aa-Induced Pro-Inflammatory Cytokine Release or Secretion

**Figure 3:**
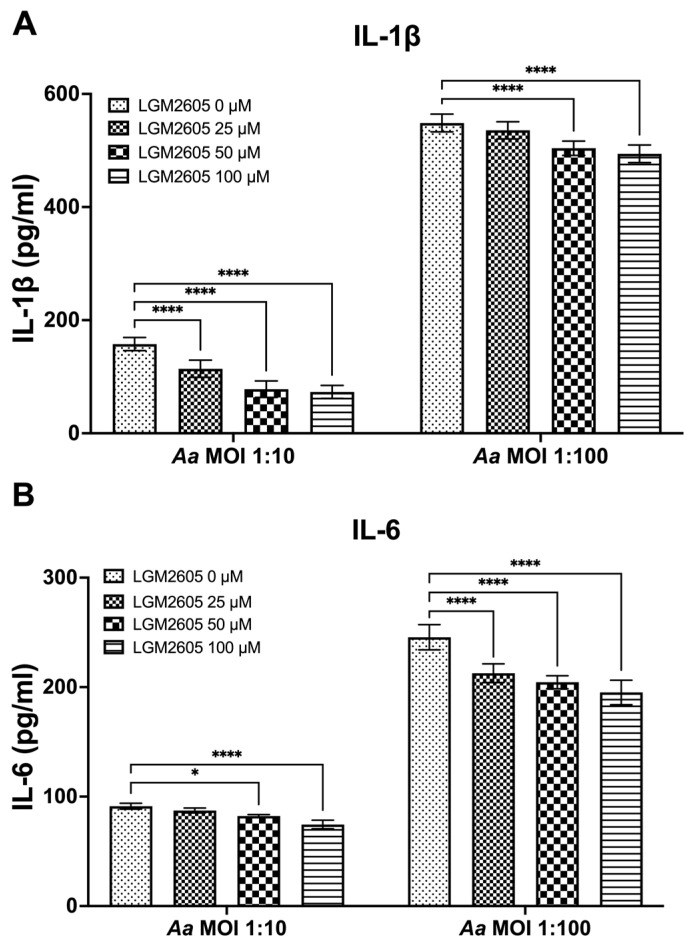

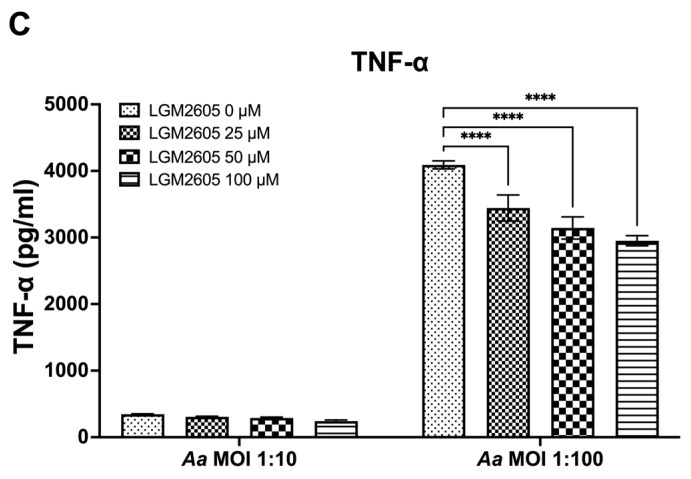
LGM2605 decreases *Aa*-induced inflammation. TDM were pre-treated with LGM2605 in increasing doses (25, 50, and 100 μM) for 30 min and inoculated with mid-log phase *Aa* (MOI of 1:10 and 1:100) for 4 h. Supernatants were collected and analyzed by ELISA specific for IL-1β (Panel (**A**)), IL-6 (Panel (**B**)), and TNF-α (Panel (**C**)). Data represent three independent experiments with technical triplicates for ELISA. Statistical significance was determined using two-way ANOVA. * denotes *p*-value < 0.05, and **** denotes *p*-value < 0.001 for 0 uM LGM2605 vs. a different dose of LGM2605 within same MOI group.

The preceding studies analyzed the impact of LGM2605 on Cdt-mediated cytokine expression and release. We also examined whether LGM2605 treatment mitigated *Aa*-induced host response. TDM were pre-treated with various doses of LGM2605 (25, 50, and 100 μM) for 30 min and subsequently exposed to *Aa* (at MOI of 1:10 and 1:100) for 4 h to elicit a pro-inflammatory cytokine response. This inoculation effectively induced pro-inflammatory cytokine release, resulting in elevated levels of IL-1β (1:10—158 ± 13 pg/mL, 1:100—551 ± 19 pg/mL, Figure 3A), IL-6 (1:10—92 ± 4 pg/mL, 1:100—244 ± 14 pg/mL, Figure 3B), and TNF-α (1:10—347 ± 10 pg/mL, 1:100—4119 ± 94 pg/mL, Figure 3C).

The pre-treatment of TDM with LGM2605 significantly reduced *Aa*-induced cytokine response (Figure 3). For IL-1β, the most substantial reduction (50%) was noted in *Aa* at an MOI of 1:10 when combined with 100 μM LGM2605 (157 ± 19 pg/mL compared to 78 ± 13 pg/mL, Figure 3A). For IL-6, the most notable reduction (20%) occurred in *Aa* at an MOI of 1:100 when treated with 100 μM LGM2605 (244 ± 14 pg/mL compared to 193 ± 14 pg/mL, Figure 3B). Lastly, for TNF-α, the most pronounced reduction (27%) was observed in *Aa* at an MOI of 1:100 when combined with 100 μM LGM2605 (4119 ± 94 pg/mL compared to 2990 ± 74 pg/mL, Figure 3C).

### 3.4. Cdt Phosphatase Activity Induced Pro-Inflammatory Environment Leads to Increase in Osteoclast Differentiation

**Figure 4:**
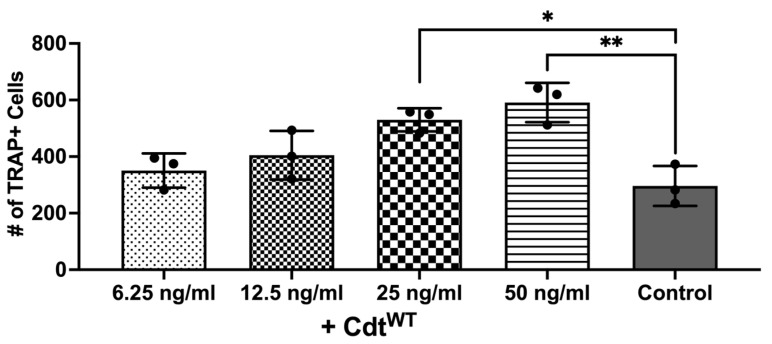
Cdt dose-dependent increase in osteoclast differentiation. THP-1 monocytes treated with PMA for 2 days and osteoclast differentiation induced with RANKL and M-CSF (50 ng/mL each) for 6 days with increasing doses of Cdt (6.25, 12.5, 25, and 50 ng/mL). At the last day, TRAP staining was conducted and TRAP + TDO were counted. Data represent three independent experiments. Statistical significance was determined using one-way ANOVA. * denotes *p*-value < 0.05 and ** denotes *p*-value < 0.01 for the control (RANKL + M-CSF only) vs. different concentration Cdt-treated groups.

The primary driver of bone breakdown is the osteoclast, the central cellular player in this process [9,10]. It stems from precursor cells of monocyte/macrophage origin, regulated by crucial cytokines such as M-CSF, RANKL, and osteoprotegerin [9,10]. But pro-inflammatory cytokines can also promote osteoclast differentiation via autocrine/paracrine mechanisms by triggering related pathways [9,10,28,29,30]. Macrophage exposure to Cdt results in PI-3K signaling blockade leading to increased cytokine release as shown above, consistent with published studies [7]. Also, increased TRAP activity was observed with Cdt-intoxicated murine osteoclasts [24,29]. Thus, we sought to determine whether Cdt can increase osteoclast differentiation in a human cell line. THP-1 cells were differentiated into macrophages with high PMA followed by osteoclast differentiation with RANKL and M-CSF in media for 6 days as described in the methods [24,29]. Increasing concentrations of Cdt were added and replenished every 2 days. TRAP staining was performed on the 6th day of differentiation and the number of TRAP+ cells were counted to analyze differentiated TDOs (THP-1 derived osteoclasts) as distinct from macrophages. An increasing trend in TRAP+ cells was observed upon increase in Cdt concentration. At 50 ng/mL Cdt, there was a two-fold statistically significant increase in TRAP+ cells (Figure 4). 

**Figure 5:**
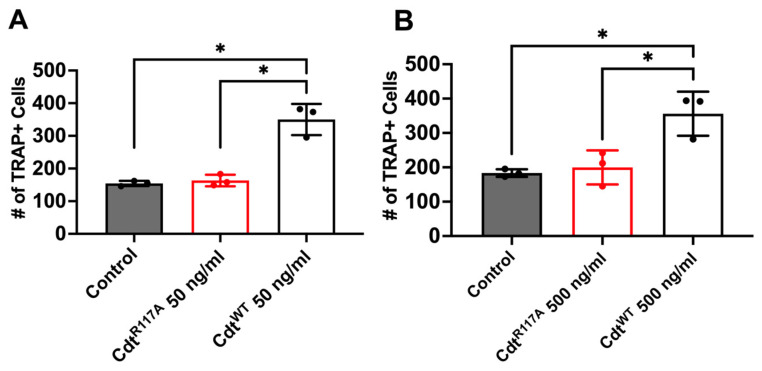
Phosphatase-dependent increase in osteoclast differentiation induced by Cdt. THP-1 monocytes underwent differentiation into macrophages using a high concentration of PMA (100 ng/mL). Osteoclast differentiation was initiated by exposing cells to RANKL and M-CSF (50 ng/mL each) for six days, with media replenishment every 48 h. Cdt treatments ((**A**) 50 ng/mL and (**B**) 500 ng/mL) for both Cdt^R117A^ and Cdt^WT^ were introduced concurrently with RANKL and M-CSF. At the endpoint, TRAP staining was conducted, and TRAP + TDO were quantified. The presented data are representative of three independent experiments. Statistical significance was determined using one-way ANOVA. * denotes *p*-value < 0.05 for the control (RANKL + M-CSF only) compared to Cdt (Cdt^R117A^ and Cdt^WT^) -treated groups.

Next, to determine whether the increase in osteoclast differentiation is dependent on CdtB phosphatase activity leading to a depletion of PI(3,4,5)P3, we utilized the phosphatase-inactive Cdt mutant (Cdt^R117A^) and wild-type Cdt (Cdt^WT^). Cells were initially treated with high PMA (100 ng/mL) for two days and differentiated to osteoclasts with RANKL and M-CSF for 6 days with Cdt^WT^, CdtR^117A^, or no addition (control). On the 6th day of differentiation, TRAP + cells were counted. First, both Cdt^WT^ and Cdt^R117A^ were tested at a concentration of 50 ng/mL based on observations in Figure 5. While a significant increase (170%, Figure 5A) in TRAP+ cells compared to control was detected with Cdt^WT^, no significant difference was noted with Cdt^R117A^ (107%, Figure 5A) compared to control. Subsequently, we explored if possible alternative mechanisms trigger differentiation beside phosphatase activity by Cdt^R117A^ by using a 10 times higher concentration (500 ng/mL, Figure 5B). Once again, Cdt^WT^-treated cells exhibited a significant increase (200%) compared to the control, whereas no significant difference (109%) was observed with Cdt^R117A^ compared to the control. These findings collectively suggest a dependence on CdtB phosphatase activity, specifically implicating PI(3,4,5)P3 depletion, in the observed enhancement of TDO differentiation.

### 3.5. Cdt-Mediated Increase in Osteoclast Differentiation/Maturation Can Be Mitigated by LGM2605

**Figure 6:**
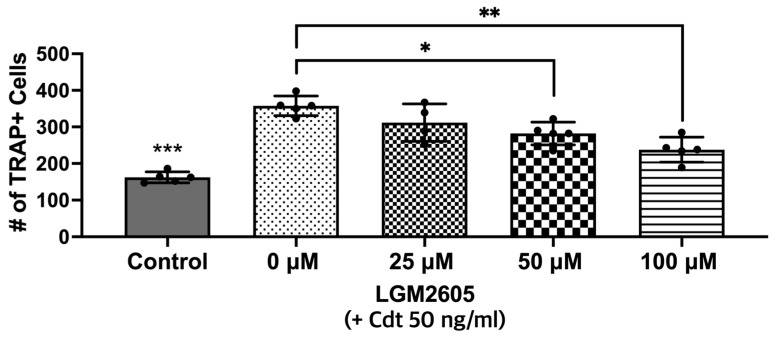
LGM2605 reduces Cdt-mediated increases in osteoclast differentiation. First, THP-1 monocytes were treated with a high PMA concentration (100 ng/mL). Before RANKL and M-CSF (50 ng/mL each) induced TDO differentiation with or without Cdt treatment (50 ng/mL) for 6 days, THP-1 cells were pretreated with different concentrations of LGM2605 (25, 50, and 100 μM) for 30 min in media. Media with RANKL and M-CSF replenishment every 48 h was used with and without Cdt and LGM2605. TRAP staining was conducted at the 6th day of differentiation and TRAP+ cells were counted for three independent experiments. Data represent three independent experiments with technical triplicates. Statistical significance was determined using one-way ANOVA. * denotes *p*-value < 0.05 and ** denotes *p*-value < 0.01 for Cdt-treated vs. Cdt + LGM2605-treated groups. *** denotes *p*-value < 0.005 for control vs. all other groups.

In this next set of experiments, LGM2605 was assessed for its ability to impair the osteoclast differentiation of Cdt-treated TDO. THP-1 cells were with LGM2605 after PMA and during TDO differentiation with media containing RANKL and M-SCF. Cdt 50 ng/mL was added for Cdt treatment and Cdt + LGM2605 treatment. TRAP staining revealed that there was a significant decrease (*p*-value < 0.05 and < 0.01, respectively) in TRAP activity when LGM2605 50 μM (79% of Cdt only) and 100 μM (66% of Cdt only) were applied concurrent with 50 ng/mL Cdt treatment during differentiation (Figure 6). 

**Figure 7:**
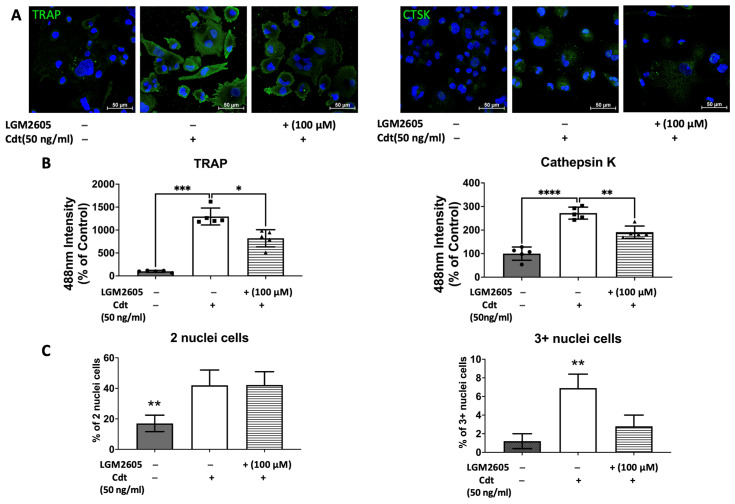
Impact of Cdt and LGM2605 on osteoclast maturation. THP-1 cells underwent treatment with a high concentration of PMA (100 ng/mL) for two days before osteoclast differentiation using RANKL and M-CSF (50 ng/mL each) for six days. PMA-treated cells were pretreated with 100 μM LGM2605 for 30 min before differentiation and Cdt (50 ng/mL) was added with differentiation media. Media replenishment with RANKL and M-CSF, with or without Cdt and LGM2605, occurred every 48 h. Immunostaining for TRAP, cathepsin K (CTSK), and nuclei was performed, followed by multi-fluor confocal imaging as detailed in the Section 2. (**A**) Maximum intensity projection images depict control (only RANKL- and M-CSF-induced differentiation), Cdt treatment (50 ng/mL), and Cdt (50 ng/mL) + LGM2605 (100 μM) treatment for TRAP (**left**) and CTSK (**right**) in green, with nuclei in blue. (**B**) Quantification of TRAP (**left**) and CTSK (**right**) fluorescence intensity presented as a percentage relative to the control. Results are expressed as mean ± STDEV (five fields per condition) and compared using one-way ANOVA. Statistical significance indicated by *, *p*-value < 0.05; **, *p*-value < 0.01; ***, *p*-value < 0.005; and ****, *p*-value < 0.001 vs. other conditions. (**C**) Percentage of two nuclei per cell (**left**) and three or more nuclei per cell (**right**) in each condition. The number of nuclei per cell was counted from confocal images and calculated as a percentage of whole population per condition. Graphs displaying cells with two nuclei per cell and three or more nuclei per cell were generated to represent maturation. Mean ± STDEV (five fields per condition) and statistical comparisons using one-way ANOVA. **, *p*-value < 0.01 vs. other conditions.

**Figure 8:**
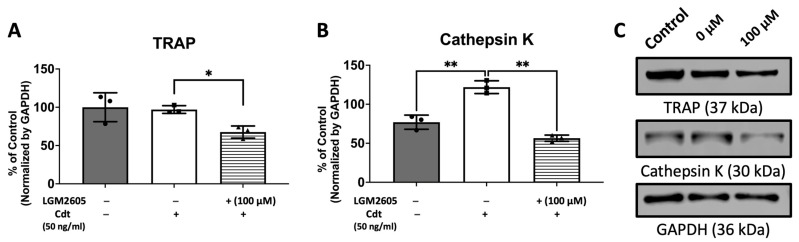
LGM2605 mitigates Cdt-induced osteoclast differentiation markers. THP-1 monocytes were initially treated with a high concentration of PMA (100 ng/mL) for two days. Subsequently, TDO differentiation was induced by RANKL and M-CSF (50 ng/mL each) for six days, with or without concurrent treatment of Cdt (50 ng/mL) after pretreating PMA-treated THP-1 cells with 100 μM LGM2605 for 30 min. Media containing RANKL and M-CSF, with or without Cdt and LGM2605, was replenished every 48 h. Lysates were collected and subjected to Western blot analysis as described in the Section 2. (**A,B**) Quantitative data depict the mean ± STDEV of TRAP (**A**) and CTSK (**B**) levels, presented as a percentage relative to the untreated control. Data represent three independent experiments and are compared using one-way ANOVA. *, *p*-value < 0.05 compared to other conditions; **, *p*-value < 0.01. (**C**) Representative immunoblot images demonstrating TRAP and CTSK staining, with GAPDH used as an internal normalization protein.

To further confirm the effect of Cdt and LGM2605 on osteoclast maturation, we examined levels of osteoclast maturation markers, TRAP and cathepsin K (capacity to degrade bone tissue), and the number of nuclei (multinucleation: the late phase of osteoclast differentiation) [9,10,28]. Cathepsin K, is one of the highly effective proteases, primarily tasked with facilitating bone resorption [9,30]. Multi-fluor confocal imaging (Figure 7) revealed a significant increase in TRAP staining (1295% of control) and cathepsin K immunoreactivity (272% of control) when 50 ng/mL Cdt was added during TDO differentiation. The addition of 100 μM LGM2605 mitigated the effect of Cdt as indicated by a significant decrease in TRAP level (820% of control) and cathepsin K level (185% of control). Osteoclast maturation is determined by multinucleation [9,10,28], and therefore we counted the number of nuclei per cell. While both the Cdt-only group (42% of cells) and Cdt+LGM2605 group (42.2% of cells) had significant increase in two nuclei per cell compared to control (17% cells), the occurrence of three or more nuclei was significantly less in the Cdt + LGM2605 group (2.8% of cells) and control group (1.2% of cells) compared to the Cdt-only group (6.9% of cells). We also analyzed protein levels of osteoclast markers by Western blot (Figure 8). TRAP levels were highest with Cdt alone (control group); there was a significant decrease in the Cdt + LGM2605 group (55% of control) compared to the Cdt-only group (77% of control). Cathepsin K revealed that the Cdt-only group had the highest level (150% of control) compared to the Cdt + LGM2605 group (89% of control) and control group.

## 4. Discussion

Our study probes the intricate interplay between Cdt, produced by *Aa* and the host’s inflammatory response. Previous studies have indicated that Cdt’s phosphatase activity disrupts PIP3 signaling, impacting the phosphorylation of GSK3β and Akt and modulating pro-inflammatory cytokine expression through NF-κB activation [5,7]. When treating macrophages with Cdt, we observed a significant upregulation of pro-inflammatory genes and corresponding cytokine release, including pro-IL-1β, IL-6, and TNF-α. These observations support the notion that Cdt actively contributes to the development of the hyper-inflammatory environment associated with MIPP, ultimately exacerbating the disease’s progression. 

Moreover, our study highlights a potential solution to mitigate the inflammatory response triggered by Cdt. We explored the application of LGM2605, a bioactive lignan known for its anti-inflammatory properties. It notably reduced the expression of pro-inflammatory genes in Cdt-exposed macrophages. LGM2605 also impaired the release of IL-1β, IL-6, and TNF-α in macrophages exposed to Cdt or challenged with *Aa*. These results echo previous studies involving LGM2605 in asbestos- and radiation-triggered lung disease, age-related retinal epithelium disease, reactive oxygen species-involved cardiovascular disease, and neuronal inflammatory disease [15,16,21,22,23]. On a molecular level, one aspect of LGM2605′s action is to inhibit NF-κB nuclear translocation while activating downstream targets, alongside augmenting Nrf2 activity and the antioxidant response [16,31,32]. In our investigations, the administration of LGM2605 during Cdt intoxication resulted in a significant reduction in the gene expression levels of pro-IL-1β (to a lesser extent), IL-6, and TNF-α (to a greater extent). However, the observed secretion of these cytokines did not exhibit the same proportioned decrease as seen in gene expression. This discrepancy might be attributed to the timing of RNA extraction and supernatant collection concerning the temporal dynamics between gene expression and protein secretion. Our assessment focused on a limited timeframe of two hours for gene expression and five hours for protein secretion. Conducting experiments in a time-course manner could elucidate more comprehensive insights into the dynamic interplay between gene expression and subsequent protein secretion. We also note that IL-1β is expressed in the pro-IL-1β form and must be proteolytically cleaved to IL-1β for subsequent secretion [27]. It is also important to note that cytokine secretion, specifically that of IL-1β, relies on gasdermin cleavage, adding complexity and likely compromising a one-to-one relationship between cytokine gene expression and secretion [27]. Given the exacerbated inflammation induced by Cdt and *Aa* infection in MIPP, LGM2605 emerges as a promising therapeutic agent to counteract the detrimental effects observed, aligning with its success in managing similar pathological conditions.

As was mentioned in the introduction, the inflammatory cytokines are capable of impacting osteoclast formation, and multiple studies, with animals and humans, have shown that *Aa* has influenced and increased osteoclast formation leading to bone loss [9,10,28,30,33,34,35]. In our investigation, we found that Cdt impacts osteoclast differentiation and maturation, a crucial aspect of MIPP pathogenesis. Our study revealed that Cdt promotes the increase in TRAP+ cells, indicating a heightened differentiation of TDO. In our study, the Cdt-induced release of inflammatory cytokines likely stimulates this differentiation process. Crucially, we established that this effect of Cdt relies on its phosphatase activity. When cells were treated with Cdt^R117A^, a phosphatase-inactive form of Cdt, there was no significant increase in TRAP+ cells, confirming the dependency of this effect on phosphatase activity. The result was consistent across concentrations of low (50 ng/mL) and high (500 ng/mL) concentrations (Figure 5). Beyond the TRAP+ assay, we assessed TDO maturation using TRAP and cathepsin K via immunoblotting and immunocytochemistry, in addition to evaluating the number of nuclei per cell.

TRAP have traditionally served as markers for osteoclast differentiation [13,14,15,28,32]. However, it is crucial to note that while TRAP is expressed and secreted by various monohistiocytic lineage cells, it does not precisely define the differentiation of cells [28,36]. In macrophages, TRAP localizes within lysosomes, while in osteoclasts, it resides in vesicles that fuse with transcytotic vesicles and are delivered to the basolateral surface, with other bone degrading enzymes, where they are secreted for bone resorption [28]. Thus, protein amount might not justify the differentiation of osteoclasts from macrophages and it is possible that the location of TRAP is more crucial to define the differentiation. This was evident in our study as well. While immunoblot analysis did not reveal a statistical difference in protein levels between the control and Cdt-treated TDO (Figure 8), our immunocytochemistry results showed significant differences. Specifically, the control group displayed a stronger TRAP intensity localized in a granular pattern with faint diffusion in the basolateral side of the cell. In contrast, the Cdt-treated TDO exhibited strong intensity across the entire basolateral side of the cell. This difference in TRAP distribution aligns with its distinct localization in macrophages and osteoclasts. 

Cathepsin K is primarily secreted by activated osteoclasts to facilitate the degradation of collagen and other matrix proteins during bone resorption [9,10]. While TRAP activity can be detected in pre-osteoclastic differentiating cells, cathepsin K serves as an osteoclast maturation marker indicative of activated osteoclasts possessing the capacity to degrade bone tissue [9,28]. In our study, both immunoblot and immunocytochemistry analyses revealed an increase in cathepsin K levels when TDOs were treated with Cdt compared to control (Figure 7 and Figure 8). Additionally, we assessed osteoclast maturation by examining the number of nuclei per cell. Although osteoclasts that fail to multinucleate can retain certain osteoclast characteristics, express markers like TRAP and cathepsin K, and maintain limited bone resorption ability [28], multinucleation is a hallmark of the late phase of osteoclast maturation [9,10,28,33]. In our study, we distinguished maturation by categorizing cells with two nuclei per cell from those with three or more nuclei per cell. The Cdt-treated TDO showed a significantly higher population in both categories, indicating a further progression in the maturation process. The collective findings suggest that the Cdt-triggered inflammatory response disrupts the balance of osteo–immune homeostasis, fostering bone loss—a defining characteristic of periodontitis.

As the treatment with Cdt increased TDO differentiation and maturation, we investigated whether LGM2605 might have the potential to counteract this effect. Given that LGM2605 significantly reduces the inflammatory response induced by Cdt and *Aa* in TDM (Figure 2 and Figure 3), we hypothesized that the dampened pro-inflammatory cytokine levels by LGM2605 could also lead to a decrease in TDO differentiation and maturation, which are known to be stimulated by such cytokines [33,34,35]. In the TRAP+ assay, while all concentrations of LGM2605 treatment exhibited a significant increase compared to the control, LGM2605 at 50 µM and 100 µM notably reduced the number of TRAP+ cells compared to conditions treated with Cdt alone (Figure 6). In the maturation study conducted through immunocytochemistry, although it did not exhibit a significant decrease in cathepsin K levels or the population of cells with two nuclei per cell, it did reveal a decrease in TRAP intensity and the population of cells with three or more nuclei per cell (Figure 7). Additionally, the maturation study employing immunoblotting demonstrated a notable decrease in both TRAP and cathepsin K levels compared to conditions treated solely with Cdt (Figure 8). LGM2605 possibly mitigated inflammatory cytokines, resulting in the suppression of TDO differentiation and maturation. This effect might also involve the activation of Nrf2. Extensive studies on primary cells or cell lines have demonstrated that Nrf2 hyperactivation inhibits osteoclast differentiation, whereas its inhibition yields contrasting effects [37,38,39]. RANKL’s binding to RANK triggers crucial intracellular signaling cascades through TRAF-6, leading to ROS generation crucial for osteoclast formation [37,38,39]. Nrf2’s regulation of oxidative stress could be a primary mechanism influencing osteoclast differentiation [37,38,39]. In this study, LGM2605 demonstrated its ability to mitigate this effect, introducing a novel approach to managing bone loss in MIPP.

While our research has provided valuable insights, it is imperative to acknowledge certain limitations that pave the way for future advancements. Initially, most experiments were conducted using THP1, a human leukemia monocytic cell line, thereby confining our study to an in vitro setting. Recognizing this limitation, further investigations are crucial to fully elucidate the impact of Cdt and LGM2605 on pro-inflammatory conditions and osteoclast differentiation/maturation in a more physiologically relevant in vivo environment. The integration of animal studies will undoubtedly fortify LGM2605’s therapeutic potential, bridging the gap between experimental and clinical applicability. Moving forward, clinical trials are imperative to rigorously assess LGM2605’s safety and effectiveness as a potential treatment for MIPP and other inflammatory diseases. In conclusion, our study has not only uncovered the intricate relationship between Cdt, the host’s inflammatory response, and osteoclast differentiation but has also illuminated crucial pathways in pathogenesis of MIPP. The potential therapeutic role of LGM2605 opens up innovative avenues for managing MIPP and other inflammatory conditions, offering renewed hope for patients. As we embark on the next phase of research, the promising findings from our study lay a robust foundation for future investigations, shaping the landscape of potential treatments.

## Figures and Tables

**Table 1:** List of antibodies.

Antibody (Host)	Source (Catalog #)	Dilution (Application)
Cathepsin K, CTSK (Rabbit)	Proteintech (11239-1-AP)	1:500 (Western Blot) 1:100 (Immunocytochemistry)
Tartrate-resistant acid phosphatase, TRAP (Rabbit)	Invitrogen (PA5-116970)	1:1000 (Western Blot) 1:100 (Immunocytochemistry)
Glyceraldehyde 3-phosphate dehydrogenase, GAPDH (Rabbit)	Cell Signaling (D16H11)	1:2500 (Western Blot)

**Table 2:** List of genes used for quantitative real-time PCR.

Gene ID	Gene Bank	Assay ID	Amplicon Length
IL1B	NM_000576.2	Hs01555410_m1	91
IL6	NM_000600.4	Hs00174131_m1	95
TNFa	NM_000594.3	Hs00174128_m1	80
GAPDH	NM_001256799.2	Hs02786624_g1	157

**Table 3:** List of abbreviations.

Abbreviation	Definition
*Aa*	*Aggregatibacter actinomycetemcomitans*
ARE	Antioxidant response element
Cdt	Cytolethal distending toxin
Cdt^R117A^	Cdt holotoxin containing phosphatase deficient CdtB subunit
Cdt^WT^	Cdt holotoxin containing the wildtype CdtB subunit
CFU	Colony forming unit
CTSK	Cathepsin K
D7S	Wild-type *Aa*
ELISA	Enzyme-linked immunosorbent assay
GCF	Gingival crevicular fluid
IL-1	Interleukin-1
IL-6	Interleukin-6
LAP	Localized aggressive periodontitis
LGM2605	SDG synthetic counterpart
M-CSF	Macrophage colony-stimulating factor
MIPP	Molar/incisor pattern periodontitis
NF-κB	Nuclear factor-κB
Nrf2	Nuclear factor (erythroid-derived 2)-like 2
OC	Osteoclast
PGE2	Prostaglandin E2
PI	Phosphatidylinositol
PIP2	Phosphatidylinsoitol-3,4-diphosphate
PIP3	Phosphatidylinositol-3,4,5-triphosphate
PMA	Phorbol 12-myristate 13-acetate
RANKL	Receptor activator of nuclear factor-κB ligand
SDG	Secoisolariciresinol diglucoside
TDO	THP-1 cell derived osteoclast
TDM	THP-1 cell differentiated macrophage
TNF-α	Tumor necrosis factor alpha
TRAP	Tartrate-resistant acid phosphatase

## Data Availability

The original contributions presented in the study are included in the article, further inquiries can be directed to the corresponding author.

## Supplementary Materials

The following supporting information can be downloaded at: https://www.mdpi.com/article/10.3390/dj12070195/s1, Figure S1. LGM2605 does not affect the cell viability.
