# Supplementary material for: A Synthetic Small Molecule, LGM2605: A Promising Modulator of Increased Pro-Inflammatory Cytokine and Osteoclast Differentiation by Aggregatibacter actinomycetemcomitans Cytolethal Distending Toxin
Source: Dent J (Basel). 2024 Jun 26;12(7):195. doi: 10.3390/dj12070195 (PMC11276599; doi:10.3390/dj12070195)
Supplement: Supplementary file 1 [file dentistry-12-00195-s001.zip › dentistry-2965310-supplementary.pdf]

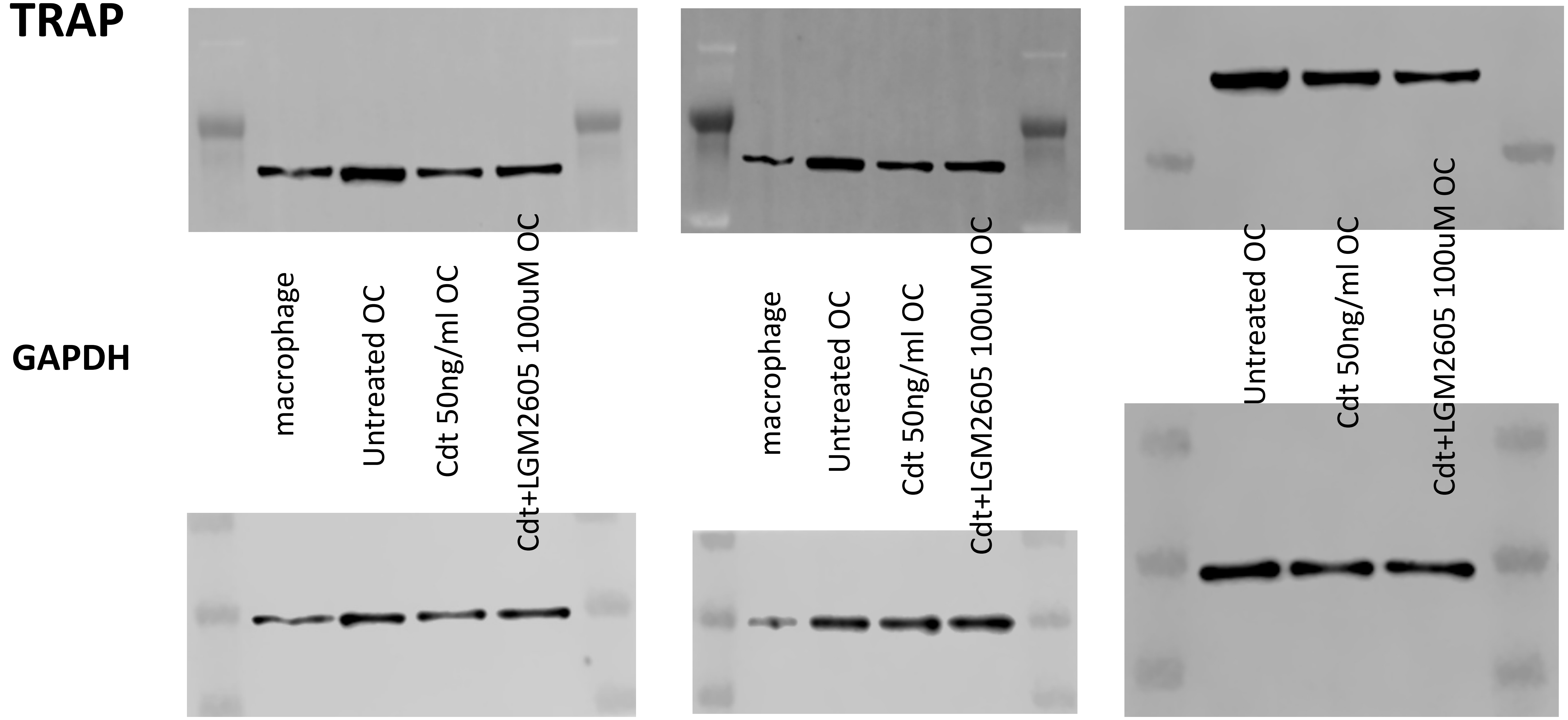

OC = Osteoclast

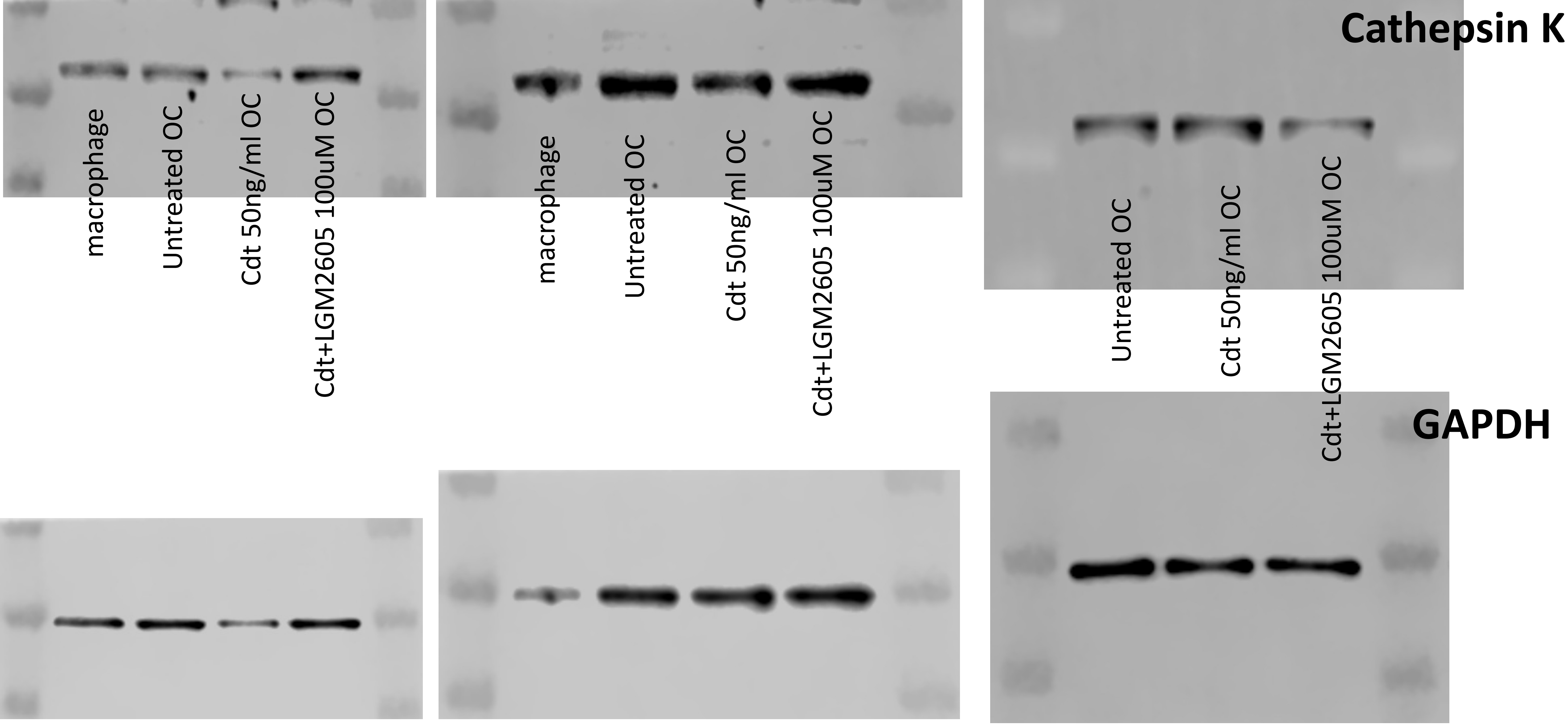

OC = Osteoclast
